# Beta-Lactams Toxicity in the Intensive Care Unit: An Underestimated Collateral Damage?

**DOI:** 10.3390/microorganisms9071505

**Published:** 2021-07-14

**Authors:** Claire Roger, Benjamin Louart

**Affiliations:** 1 Department of Anesthesiology and Intensive Care, Pain and Emergency Medicine, Nîmes-Caremeau University Hospital, Place du Professeur Robert Debré, CEDEX 9, 30029 Nîmes, France; benjamin.louart@chu-nimes.fr; 2 UR UM 103 IMAGINE, Faculty of Medicine, Montpellier University, 34090 Montpellier, France

**Keywords:** antimicrobials, nephrotoxicity, neurotoxicity, adverse events, critically ill patients

## Abstract

Beta-lactams are the most commonly prescribed antimicrobials in intensive care unit (ICU) settings and remain one of the safest antimicrobials prescribed. However, the misdiagnosis of beta-lactam-related adverse events may alter ICU patient management and impact clinical outcomes. To describe the clinical manifestations, risk factors and beta-lactam-induced neurological and renal adverse effects in the ICU setting, we performed a comprehensive literature review via an electronic search on PubMed up to April 2021 to provide updated clinical data. Beta-lactam neurotoxicity occurs in 10–15% of ICU patients and may be responsible for a large panel of clinical manifestations, ranging from confusion, encephalopathy and hallucinations to myoclonus, convulsions and non-convulsive status epilepticus. Renal impairment, underlying brain abnormalities and advanced age have been recognized as the main risk factors for neurotoxicity. In ICU patients, trough concentrations above 22 mg/L for cefepime, 64 mg/L for meropenem, 125 mg/L for flucloxacillin and 360 mg/L for piperacillin (used without tazobactam) are associated with neurotoxicity in 50% of patients. Even though renal complications (especially severe complications, such as acute interstitial nephritis, renal damage associated with drug induced hemolytic anemia and renal obstruction by crystallization) remain rare, there is compelling evidence of increased nephrotoxicity using well-known nephrotoxic drugs such as vancomycin combined with beta-lactams. Treatment mainly relies on the discontinuation of the offending drug but in the near future, antimicrobial optimal dosing regimens should be defined, not only based on pharmacokinetics/pharmacodynamic (PK/PD) targets associated with clinical and microbiological efficacy, but also on PK/toxicodynamic targets. The use of dosing software may help to achieve these goals.

## Key Points

- The risk of neurotoxicity differs widely among beta-lactam antibiotics.
- The occurrence of a neurological and/or psychiatric sign in patients treated with beta-lactams, especially in the event of renal insufficiency or underlying brain abnormalities, should lead physicians to suspect some degree of neurotoxicity.
- High beta-lactam exposure is common in ICU patients and is associated with an increased risk of neurotoxicity.
- Nephrotoxicity related to beta-lactams is mainly characterized by the occurrence of acute interstitial nephritis, renal damage associated with drug induced hemolytic anemia and renal obstruction by crystallization.
- Therapeutic drug monitoring and well-defined toxicity thresholds may help in the future to better tailor beta-lactam dosing regimens in ICU patients.

## 1. Introduction

Beta-lactams are widely used as a first-line therapy because they provide broad-spectrum coverage of different pathogens and display bactericidal properties and few drug-related complications. Beta-lactams are commonly considered safe drugs but adverse effects are classically reported [1,2]. Although these antimicrobials are essential in order to treat severe infections, their overuse and unnecessary prolonged administration may become harmful via different mechanisms, including mitochondrial toxicity, adverse drug reactions and the selection of resistant organisms, as well as disruption of the microbiome.

However, as monitoring of beta-lactam plasma concentrations has become increasingly available, the risk of over-exposure is now better identified in critically ill patients. Nevertheless, the need for higher-than-conventional dosing regimens for less susceptible strains and the reduction in the glomerular filtration rate may lead to accumulation and adverse events. There is compelling evidence that toxicity occurs in intensive care unit (ICU) patients even with beta-lactams [2]. Previous reports describing beta-lactam toxicity have been published but none of them has focused on neurotoxicity and nephrotoxicity in the setting of ICUs, where the diagnosis of toxicity may be compromised [3,4].

This article aims to review the most common and important neurotoxic and nephrotoxic adverse events associated with beta-lactam antibiotics routinely used in the ICU setting. It provides a clear and practical overview, enabling professionals to better identify and manage such complications in adult ICU patients. Finally, it discusses approaches to limit beta-lactam-related neurological and renal toxicities and highlights research priorities.

## 2. Literature Search

We identified all the studies through a literature search of electronic databases, including the MEDLINE database and Google scholar for studies published between 1980 and 2021. The search terms were: “Beta-lactam toxicity” OR “Beta-lactam and toxicity”, “Beta-lactam Neurotoxicity” OR “Beta-lactam and Neurotoxicity” AND “Seizures” “Encephalopathy”, “Beta-lactam Nephrotoxicity” OR “Beta-lactam and Nephrotoxicity”, “Beta-lactam and Acute Interstitial nephritis”. All review articles, case reports, and other relevant data were enrolled in the study after a review and agreement by two of the authors was obtained. Finally, 74 publications were enrolled in this narrative review.

## 3. Beta-Lactam Neurotoxicity

Neurotoxicity refers to the capability of inducing adverse effects in the central nervous system (CNS), peripheral nerves, or sensory organs. In recent decades, the adverse effects of beta-lactam antibiotics on the CNS have become more widely recognized. Beta-lactam overexposure and high plasma concentrations due to unadjusted dosing regimens in renally impaired critically ill patients have been frequently implicated in neurological adverse reactions [5,6].

### 3.1. Physiopathology

**Figure 1:**
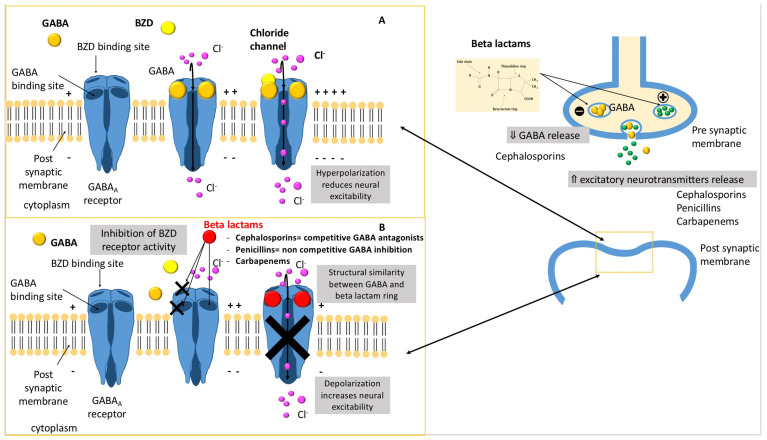
Schematic representation of the different mechanisms involved in beta-lactam-induced neurotoxicity. (**A**) GABA_A_ receptor function, (**B**) beta-lactam neurotoxicity mechanisms.

The underlying mechanism of beta-lactam neurological adverse drug reactions is not fully well known. Beta-lactams cause central excitotoxicity through a variety of mechanisms [7,8,9] (Figure 1):

- A decrease in gamma-aminobutyric acid (GABA) neuroinhibitory tone through the concentration-dependent inhibition of subunits of the GABA_A_ receptor complex in a competitive (cephalosporins) or non-competitive (penicillins) way. Indeed, the activation of GABA_A_ receptor by endogenous GABA results in an intracellular influx of chloride ions, creating an inhibitory postsynaptic potential that increases the threshold for the generation of an action potential.
- A decrease in the GABA release from nerve terminals.
- The inhibition of the activity of benzodiazepine receptors.

Notably, positive modulators of GABA_A_ receptors, such as benzodiazepines and barbiturates, are more efficacious than phenytoin to treat convulsions induced by beta-lactam antibiotics [10]. This observation gives credence to the role of GABA_A_ receptors in antibiotic-induced neurotoxicity.

- Direct antagonistic action at the GABA_A_ receptor complex.

Beta-lactam antibiotics can bind directly to the GABA_A_ receptor due to the structural similarity between GABA and the beta-lactam ring. Thus, the beta-lactam ring structure is an important determinant of its epileptogenic properties. The cleavage of this ring with penicillinase abolishes the excitatory effects of penicillin applied directly to the cortex in vivo [11]. GABA_A_ channels can open in the absence of GABA, but penicillin holds the GABA_A_ receptor in an open conformation and prevents ion conduction until it is removed [12].

- Additionally, cytokine and endotoxin releases induced by cephalosporins have been incriminated in neurotoxic mechanisms [13].
- Finally, antimicrobials may directly affect human mitochondrial function and may contribute to the mitochondrial dysfunction and associated organ failure in sepsis [3].

### 3.2. Clinical Manifestations

The diagnosis of neurotoxicity is highly challenging in intensive care unit (ICU) patients as no specific sign exists. A large panel of clinical signs has been reported, ranging from encephalitic signs, comprising confusion, disturbed vigilance, encephalopathy and hallucinations, to abnormal movement disorders, such as asterixis, dyskinesia, myoclonus, convulsions and status epilepticus, according to previous studies and review articles [1,14,15]. Additionally, psychiatric symptoms can develop in the form of recurrent panic attacks, depressive syndrome or post-traumatic stress disorder, especially in patients with a specific psychological context receiving penicillin administration [16]. The time to onset of these clinical manifestations is highly variable according to the beta-lactam considered and the clinical setting, ranging from 24 h to 30 days [1].

### 3.3. Electroencephalogram (EEG)

The electroencephalogram may show paroxysmal abnormalities such as diffuse spike waves, sharp waves, slow waves or diffuse triphasic waves and diffuse slow activity. Non-convulsive-status epilepticus has been also reported. In two recent systematic reviews, approximately 25% to 30% of the patients had non-convulsive status epilepticus [17,18]. In a retrospective study including 42 patients receiving cefepime and undergoing EEG, generalized periodic discharge with or without triphasic morphology was the most common EEG pattern (38%), followed by generalized rhythmic delta activity (26%) and generalized spike-and-wave patterns (10%) [19]. Additionally, focal seizures, suggesting the possibility of EEG lateralization in cefepime-induced neurotoxicity, have been described. In critically ill patients receiving sedatives, such clinical or EEG features are highly confounding and require imaging investigations in order to rule out alternative clinical diagnoses.

### 3.4. Prevalence of Neurotoxicity

The recognition of beta-lactam-induced neurotoxicity may be impeded in patients with alternative causes of brain dysfunction and its prevalence is likely to be underestimated. In related studies, neurotoxicity has been reported in up to 10–15% of ICU patients and is associated with significantly higher beta-lactam trough concentrations [14]. Toxicity has not been universally linked to higher drug concentrations, however. Analyzing different beta-lactams, a standardized (i.e., considering the clinical breakpoint of *P. aeruginosa* of 8 mg/L for cefepime, 16 mg/L for piperacillin tazobactam and 2 mg/L for meropenem) minimal concentration/minimal inhibitory concentration (Cmin/MIC) ratio >8 has been correlated with an incidence of neurological deterioration up to 60% [20].

### 3.5. Neurotoxicity Risk Factors

#### 3.5.1. Variable Risk According to Beta-Lactam Molecules

Neurological adverse reactions due to beta-lactams were first described after the intraventricular administration of penicillin G [21,22]. Significant differences in neurotoxic potential have been reported for various beta-lactams (Table 1). Differences in blood–brain barrier penetration (benzylpenicillin 2%, cefazolin 0.7–10%, cefepime 10%, imipenem 20%) and underlying mechanisms of toxicity observed among beta-lactam drugs may partially explain the variable risk of neurotoxicity [23]. Piperacillin has previously been implicated in neuropsychiatric manifestations, especially in critically ill patients, due to significantly altered PK factors.

Among the cephalosporins, molecules such as cefepime and cefazolin have a lower neurotoxicity threshold and are more strongly associated with seizure-triggering properties than other beta-lactam antibiotics [1,18,24,25]. However, the use of cefazolin as a surgical antibiotic prophylaxis has not been associated with neurological events, suggesting that longer exposure increases the risk of neurotoxic adverse events. A literature review including 37 studies, representing 135 patient cases of neurotoxicity related to cefepime administration, showed that cefepime neurotoxicity occurred in 48% of cases in patients who overdosed, but in 26% of cases in patients who were appropriately dosed, taking into account their renal function [18]. Additionally, several retrospective studies have incriminated cefepime in the occurrence of encephalopathy [14,26]. The development of non-convulsive status epilepticus has been observed following treatment with cefepime, despite normal renal profiles.

Due to their structural differences, the risk of neurotoxicity differs between various subclasses of carbapenems. For example, it has been shown that due to differences in the C-2 side chain, meropenem is less neurotoxic than imipenem [24,27]. A very low incidence of seizures in patients with or without meningitis treated with meropenem was reported, demonstrating the good CNS tolerability of this carbapenem. In a meta-analysis of randomized controlled trials, the odds ratios (ORs) for the risk of seizures from imipenem, meropenem, ertapenem and doripenem compared with non-carbapenem antibiotics (mostly third-generation cephalosporins, aminoglycosides and fluoroquinolones) were 3.50 (95% CI 2.23, 5.49), 1.04 (95% CI 0.61, 1.77), 1.32 (95% CI 0.22, 7.74) and 0.44 (95% CI 0.13, 1.53), respectively [28]. However, although the risk of seizures in the combined carbapenem arms was significantly higher than in non-carbapenem comparator arms (OR 1.87, 95% CI 1.35, 2.59), no significant difference in seizure risk between imipenem and meropenem was found in the head-to-head comparison [28]. Notably, the concomitant administration of carbapenem and valproic acid decreases the valproic acid concentration between 58% and 88.7%, with an increase in its clearance of 191% and a decrease in its half-life between 50% and 80% [29]. This mechanism could partially explain the higher risk of seizures in epileptic patients treated with imipenem and valproic acid.

In a large-scale pharmacovigilance analysis, the novel beta-lactam/beta-lactamase inhibitor combinations were found to be associated with a 10% to 14% rate of neurological adverse events, which was considered to be overestimated after deduplication. The neurotoxicity related to these agents may be related to the common use of higher dosages to improve efficacy in severe multidrug-resistant infections in ICU settings [30].

#### 3.5.2. Renal Impairment

The main risk factor associated with the neurological toxicity of beta-lactam antibiotics is renal failure, which may cause the rapid and significant accumulation of beta-lactams. A higher incidence of beta-lactam-induced CNS side effects has been observed when the beta-lactam dose was not adjusted adequately in relation to impaired renal function or to sepsis-associated glomerular filtration rate changes [31,32,33]. Reduced creatinine clearance and excess dosing of beta-lactam have been described as independent risk factors for neurotoxic effects for several beta-lactam classes. Analyzing data from 1754 patients treated with imipenem/cilastatin in phase III dose-ranging studies, Calandra et al. found that unadjusted imipenem/cilastatin dosing, particularly in patients with renal insufficiency, was associated with an increased risk of seizures [34]. Similarly, patients with severe renal dysfunction receiving higher-dose cefepime (>4 g over 48 h) have been identified as patients at greater risk of cefepime-induced neurotoxicity [35].

The pathogenesis of neurotoxicity in renally impaired patients seems to be mediated by increased beta-lactam trough concentrations, increased permeability of the blood–brain barrier secondary to a blood urea increase and the accumulation of toxic organic acids within the cerebrospinal fluid [36].

#### 3.5.3. Underlying Brain Abnormalities

Parkinson’s disease, stroke, paranoid schizophrenia treated with electroconvulsive therapy and hepatic encephalopathy have been identified as risk factors for beta-lactam-induced neurotoxicity [13].

#### 3.5.4. Advanced Age

Elderly patients present an increased risk of adverse drug events due to pharmacokinetic changes related to advanced age. Advanced age appears to be a risk factor for both neuropsychiatric events and seizure activity in patients receiving beta-lactams, especially carbapenems and piperacillin tazobactam [37].

## 4. Perspectives to Limit Beta-Lactam Neurotoxicity: Therapeutic Drug Monitoring

Therapeutic drug monitoring (TDM) has been increasingly used in the past decade to guide antimicrobial drug dosing [38,39,40]. TDM has mainly been applied to ensure maximal therapeutic antimicrobial exposure, given the high pharmacokinetic variability observed in ICU patients [41,42]. Protocols using dose adjustments based on a trough concentration taken at a steady state (between 24–48 h after treatment onset) or beta-lactam Bayesian dose adjustments have been applied. On the one hand, dose-dependent beta-lactam neurotoxicity may limit dose escalation. However, the threshold concentrations for dose-dependent toxicity are generally high, allowing the use of higher empirical dosing regimens that can be subsequently refined with TDM. Recently, some studies have demonstrated that TDM could help in minimizing toxicity for some antimicrobial agents [43]. In a retrospective study including 93 patients, no excessive drug toxicity associated with TDM-guided higher-than-licensed doses was found for either meropenem or piperacillin tazobactam, although mean daily doses were more than 40% higher in the high-dose groups [44]. However, the main barrier to widely implementing TDM-based dosing adjustments to limit toxicity is the lack of well-established thresholds for beta-lactams. A strong correlation between the occurrence of seizures and the dose of beta-lactams directly injected into brain ventricles has been reported in animal models [24]. Some studies have focused on the concentration–neurotoxicity relationship of beta-lactams in the intensive care setting. Cefepime trough concentrations above 22 mg/L (when administered by discontinuous infusions) or concentrations at a steady state above 35 mg/L (when administered by continuous infusion) have been associated with neurotoxicity in 50% of patients [26,45]. Comparatively, the same risk has been reported for troughs above 64 mg/L for meropenem, 125 mg/L for flucloxacillin and 360 mg/L for piperacillin (used without tazobactam) [6]. In combination with tazobactam, a plasma steady-state concentration of piperacillin above 157 mg/L is predictive of the occurrence of neurological disorders in ICU patients with a specificity of 97% and a sensitivity of 52% [33]. Finally, when considering a standardized MIC such as the Eucast clinical breakpoint for *P. aeruginosa*, an *f*Cmin/MIC*_P. aeruginosa_* ratio exceeding eight is associated with a significant deterioration of the neurological status occurring in approximately half of the ICU patients treated with piperacillin/tazobactam and approximately two-thirds of the ICU patients treated with meropenem [20]. As a result, the benefit–risk balance most likely decreases as *f*Cmin exceeds eight times the MIC [46] (Table 2). Most studies have attempted to define neurotoxicity thresholds using beta-lactam trough concentrations (Cmin). However, single trough concentrations may not accurately predict total antibiotic exposure. Total exposure expressed as area under the curve (AUC) should be considered in further studies to determine toxicity thresholds.

Finally, the impact of TDM-guided dosing adjustment on clinical outcomes has yet to be determined.

## 5. Beta-Lactam Nephrotoxicity

Although beta-lactam antibiotics are considered to be moderately nephrotoxic, renal damage associated with the use of this class of antibiotics has been classically reported in the literature. The causal relationship between beta-lactam use and kidney dysfunction remains difficult to prove, particularly due to many potential confounding factors, such as co-morbidities, sepsis and associated nephrotoxic drugs. Additionally, the delay in the onset of toxic clinical signs, together with variations in defining nephrotoxicity, make inferences difficult. Nephrotoxicity is often limited to acute renal failure, defined by biological parameters, which may lack sensitivity and specificity, and may only diagnose significant impairments of renal function [47]. Retrospective data investigating the incidence of acute renal failure among patients receiving beta-lactams report rates ranging from 0.15% to 50% [6,47,48]. Nevertheless, although it may be rare, clinicians, should be aware of beta-lactams contributing to nephrotoxicity, as the diagnosis can be highly challenging. Discontinuing the offending antibiotic may be essential and is often the main therapeutic strategy to apply. The most reported nephrotoxic events in the literature are represented by acute interstitial nephritis, renal damage associated with drug-induced hemolytic anemia and renal obstruction by crystallization [4]. It is also important to consider the growing body of evidence on increased nephrotoxicity when combining some known nephrotoxic drugs with beta-lactams [49].

### 5.1. Acute Interstitial Nephritis

Antibiotics are considered to be the main cause of drug-induced interstitial nephritis, mostly related to beta-lactams [50]. A large amount of cases are reported in the literature and almost all beta-lactam drugs are involved, although acute interstitial nephritis is most frequently reported with penicillins and cephalosporins [50,51]. Drug-induced interstitial nephritis is a dose-independent toxicity characterized by tubular and interstitial inflammation, resulting from a non-IgE-mediated hypersensitivity reaction involving lymphocyte T and associated with a systemic inflammatory response. The onset time ranges from a few days to a few weeks and is potentially shorter in the case of pre-exposure [52]. Patients almost always present with fever and often a skin rash. Renal impairment consists in non-oligoanuric acute renal failure with microscopic hematuria and tubular proteinuria (low-molecular-weight proteins) for which up to a quarter of patients may require dialysis [52,53]. Renal biopsy seems essential in order to confirm the diagnosis [52,53,54]. Despite the lack of strong evidence, corticosteroids are almost always given [50,54,55,56,57]. Delayed corticosteroid administration may be associated with a poorer renal prognosis, suggesting that this therapy should be considered at an early stage and the need for randomized studies in this context [54,56].

### 5.2. Nephropathy Associated with Hemolytic Anemia

Drug-induced hemolytic anemia, although rare (its estimated incidence is about 1 per million per year), is an entity that is worth investigating because of its life-threatening potential and the prognostic importance of detecting this adverse event as early as possible. The two molecules most frequently associated with hemolytic anemia are piperacillin and ceftriaxone, but almost all beta-lactams have been incriminated in hemolytic anemia [58]. The physiopathology of drug-induced hemolytic anemia is increasingly well known and has been described in detail elsewhere. It is mostly an immunologic phenomenon with drug-dependent or drug-independent drug-induced antibodies, but a non-immunologic pathway has also been described [59]. Depending on the mechanisms involved, the clinical presentation, severity and biological data may be different [59]. The time to onset may vary from a few hours, especially in children with severe clinical presentations, to several days. Its clinical features are characterized by a sharp decrease in hemoglobin, leading to organ failure and severe complications such as shock, circulatory arrest, organ ischemia, disseminated intravascular coagulation and acute respiratory distress syndrome with a potentially high mortality rate up to 30–50% [60,61]. Children present with more severe and quickly-occurring clinical features associated with worse prognosis [58,59,60]. An earlier, less obvious episode is often found to have occurred, and subsequent contact with the offending molecule is responsible for more severe symptoms [59,62]. The incidence of renal impairment in this setting could be high, around 50%. Renal failure is not only due to hypoperfusion and ischemia induced by the hemoglobin decrease and shock, but also due to the nephrotoxicity of free hemoglobin and hemin [63,64]. Discontinuation of the drug is the most important treatment measure. Patients are often given steroids although there is no proven benefit, particularly with drug-dependent antibodies [65,66]. High-dose intravenous immunoglobulins, plasmapheresis/plasma exchange and complement inhibitors have been successfully used, on the basis of theoretical pathophysiological data. However data are lacking to support these strategies [67,68]. They can be discussed according to the mechanism involved and the severity of the condition. Once the responsibility of the molecule is established, it should be contraindicated for life. The use of antibiotics of the same class should be cautious, as cross-reactions have been described [68].

### 5.3. Crystal (Obstructive) Nephropathy

Some antimicrobials precipitate as crystals in the urinary system, leading to damage in the tubular epithelium, obstruction of renal tubules and urolithiasis [69,70]. Pathophysiological mechanisms leading to kidney injury are mechanical but also inflammatory processes [70,71,72]. Most often, crystal nephropathy leads to acute kidney injury associated with hematuria, but chronic kidney disease can be observed [70]. Among beta-lactams, amoxicillin, especially when high-dose regimens are employed, is classically described, but cases of renal lithiasis have also been reported with ceftriaxone [73,74,75,76,77]. High dosing, dehydration and a urinary pH that is too acidic may promote crystalluria. Dose reduction or slowing the rate of infusion, as well as hydration, would help to prevent the risk of crystal formation.

### 5.4. AKI and Drug Association

It appears to be well established in the literature that piperacillin-tazobactam, in combination with vancomycin, is associated with an increased risk of renal failure [78,79]. The underlying pathophysiology is still unclear and the two main mechanisms suggested are acute interstitial nephritis and decreased tubular secretion of vancomycin. A meta-analysis found an odds ratio (OR) of 3.12 (95% CI 2.04–4.78) for the vancomycin–piperacillin/tazobactam combination, compared with other beta-lactams essentially in a non-ICU population [78]. More recently, a cohort study involving 2492 ICU patients found a higher incidence of acute renal failure when piperacillin-tazobactam rather than cefepime or meropenem was associated with vancomycin [36]. A retrospective study investigating burn patients also found a significantly higher incidence of acute renal failure in patients receiving the piperacillin-tazobactam/vancomycin combination rather than vancomycin alone or the imipenem/vancomycin combination [80]. If piperacillin/tazobactam is clearly associated with an increased risk of renal impairment when combined with vancomycin, to our knowledge, no study has found a significant difference in terms of mortality or chronic renal failure.

## 6. Other Beta-Lactam-Related Adverse Events

**Figure 2:**
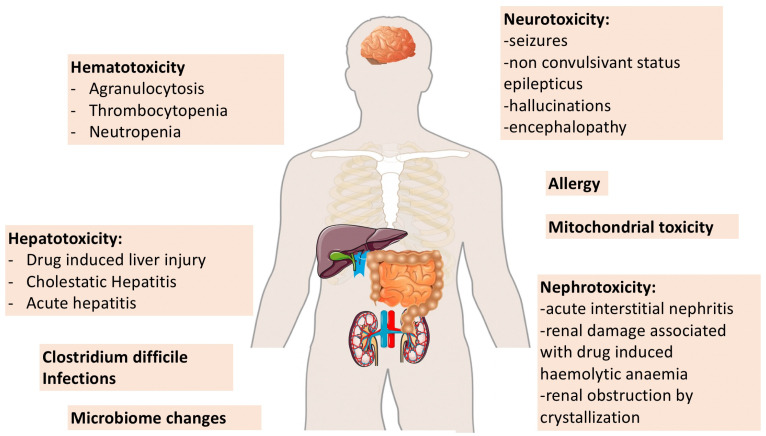
Schematic representation of different forms of collateral damage attributed to beta-lactams.

Additional examples of collateral damage caused by beta-lactams in ICU patients, such as hematological adverse events, drug-induced liver injury, allergy and *Clostridium difficile* infections, have been reported, but were not included in the present review [3,4] (Figure 2).

## 7. Future Perspectives

In the future, studies investigating the prevalence of neurotoxicity in ICU patients, including the different neurological features reported in the literature, should be carried out in order to better identify subgroups of patients at high risk of neurological adverse events. As the preponderance of evidence suggests that excessive dosing or exposure to supratherapeutic beta-lactam concentrations potentiates the risk for neurotoxicity, TDM-guided and/or software-based individualized dosing strategies should be evaluated as a means of reducing the incidence of beta-lactam-related toxicity. Nevertheless, toxicity thresholds for most beta-lactams have to be well-defined. Finally, there is a lack of data for newly commercialized beta-lactam/beta-lactamase inhibitor combinations and their related toxicities, which need to be evaluated in the ICU population in order to better determine the appropriate dosing regimens in further studies.

## 8. Conclusions

The onset of disturbed vigilance, myoclonus and/or seizure in a patient taking beta-lactam antibiotics, especially if associated with renal insufficiency or underlying brain abnormalities, should lead physicians to suspect adverse drug reactions. Routine continuous EEG and beta-lactam TDM could help to improve the diagnosis of beta-lactam-related neurotoxicity. Discontinuation of the offending drug is the best approach to retrospectively diagnose beta-lactam-induced neurotoxicity. Awareness of the potential neurotoxic clinical manifestations of beta-lactam antibiotics and enhanced vigilance in critically ill patients is essential in identifying the potentially serious, though reversible complications of beta-lactam therapy, particularly with the advent of newer antimicrobial agents. Similarly, the combination of several nephrotoxic drugs (vancomycin, piperacillin, aminoglycoside) should be used carefully, especially in patients presenting pre-existing kidney disease, older patients or patients with septic shock.

Regarding the use of antibiotics in “at risk” patients (renal dysfunction, elderly patients or patients with CNS abnormalities), clinicians should consider the benefit–risk ratio for efficacy/toxicity, especially when no alternative exists. Where possible, TDM may help to minimize toxicity, but exposure thresholds should be clearly identified.

## Figures and Tables

**Table 1:** Convulsing activity of beta-lactams compared to penicillin G [14,24].

Beta-Lactam	Relative Pro-Convulsive Activity (Reference: Penicillin G = 100)
Cefazolin	294
Cefepime	160
*Penicillin G*	*100*
Imipenem	71
Aztreonam	42
Ampicillin	21
Ceftazidime	17
Meropenem	16
Ceftriaxone	12
Piperacillin	11
Cefotaxime	8.8
Cefoxitine	1.8

**Table 2:** Suggested beta-lactam toxicity thresholds and main clinical manifestations of neurotoxicity according to the beta-lactam agent considered.

Beta-Lactam	Toxicity Threshold	Clinical Signs
Flucloxacillin [6]	Cmin > 125.1 mg/L	Seizures
		Confusion
		Myoclonia
Amoxicillin [46]	Css < 8 × MIC	Psychotic symptoms
Ceftazidime [46]	Css < 8 × MIC	Encephalopathy
		Confusion, disturbed vigilance
Cefepime [45]	Cmin > 20 mg/L	Encephalopathy
		Confusion, disturbed vigilance
Piperacillin tazobactam [6,20,33]	Css > 157.2 mg/L (pip taz CI) Cmin > 64 (pip taz)–361(pip alone) mg/L	Seizures
		Hallucinations
Imipenem [46]	Css < 8 × MIC	Seizures
		Confusion
		Myoclonia
Meropenem [6]	Cmin > 64 mg/L	Seizures

## Abbreviations

ICU	Intensive care unit
CNS	Central nervous system
GABA	Gamma-aminobutyric acid
EEG	Electroencephalography
MIC	Minimal inhibitory concentration
TDM	Therapeutic drug monitoring
